# Mild Primary or Breakthrough SARS-CoV-2 Infection Promotes Autoantibody Production in Individuals with and without Neuro-PASC

**DOI:** 10.4049/immunohorizons.2400033

**Published:** 2024-08-26

**Authors:** Lavanya Visvabharathy, Neda Dalil, Lucia Leonor, Chengsong Zhu, Zachary S. Orban, Millenia Jimenez, Patrick H. Lim, Pablo Penaloza-MacMaster, Igor J. Koralnik

**Affiliations:** * Davee Department of Neurology, Feinberg School of Medicine, Northwestern University, Chicago, IL; † Genomics and Microarray Core Facility, University of Texas Southwestern Medical Center, Dallas, TX; ‡ Department of Microbiology-Immunology, Feinberg School of Medicine, Northwestern University, Chicago, IL

## Abstract

Patients with long COVID can develop humoral autoimmunity after severe acute SARS-CoV-2 infection. However, whether similar increases in autoantibody responses occur after mild infection and whether vaccination prior to SARS-CoV-2 breakthrough infection can limit autoantibody responses is unknown. In this study, we demonstrate that mild SARS-CoV-2 infection increases autoantibodies associated with rheumatic autoimmune diseases and diabetes in most individuals, regardless of vaccination status prior to infection. However, patients with long COVID and persistent neurologic and fatigue symptoms (neuro-PASC) have substantially higher autoantibody responses than convalescent control subjects at an average of 8 mo postinfection. Furthermore, high titers of systemic lupus erythematosus– and CNS-associated autoantibodies in patients with neuro-PASC are associated with impaired cognitive performance and greater symptom severity. In summary, we found that mild SARS-CoV-2 primary and breakthrough infections can induce persistent humoral autoimmunity in both patients with neuro-PASC and healthy COVID convalescents, suggesting that a reappraisal of mitigation strategies against SARS-CoV-2 is warranted to prevent transmission and potential development of autoimmunity.

## Introduction

More than 110 million people in the United States have been infected with SARS-CoV-2, and of these, ∼30% of survivors report ever having postinfectious sequelae of SARS-CoV-2 infection (PASC), or long COVID (1), and 11% report persistent symptoms at 6 mo (2). Patients frequently complain of fatigue, myalgia, brain fog, and other neurologic sequelae as the primary drivers of decreased quality of life. Although mRNA vaccines against SARS-CoV-2 have been extremely effective in preventing severe acute disease, the incidence of PASC has not significantly decreased in the United States, despite widespread vaccine uptake (3). This indicates that PASC will remain a medical concern for the foreseeable future.

Studies have found associations between severe acute COVID-19 and subsequent autoimmunity. Hospitalized patients with COVID-19 were significantly more likely to exhibit autoantibody responses against cytokines, chemokines, and immune coreceptors (4) than those with mild disease. Similarly, evidence of anti-nuclear autoantibodies 2–3 mo after acute infection in hospitalized patients was predictive of PASC symptoms (5). The common thread between these studies lies in the association of elevated autoantibody responses with severe acute COVID-19. However, we and others have demonstrated that PASC occurs just as frequently in patients with mild acute disease (6), and it has been suggested that SARS-CoV-2 vaccines can decrease the severity of long COVID, albeit moderately (7). This brings up two critical questions: Does humoral autoimmunity contribute to PASC symptoms in patients who experienced mild acute COVID-19, and do patients with PASC infected after vaccination (breakthrough infection) have lower autoantibody responses?

In this study, we longitudinally tracked vaccine-elicited and autoantibody responses in patients with PASC with primarily neurologic symptoms (neuro-PASC) and COVID convalescent control subjects (CC) without persistent symptoms. Our data show three key findings linking SARS-CoV-2 infection, autoantibody responses, and neuro-PASC. First, mild acute infection results in vigorous increases in autoantibody titers in both patients with neuro-PASC (NP) and, to a lesser degree, CC. Second, Abs linked to rheumatic autoimmunity and neuronal dysfunction are enriched in NP, and levels are similar in patients with and without breakthrough infections. Third, elevations in anti-cytokine and anti-nuclear autoantibodies in NP are strongly associated with cognitive dysfunction and increased severity of neurologic symptoms. Together, these data show that mild SARS-CoV-2 infection results in sustained elevation of autoantibodies in both NP and, to a lesser extent, those without persistent symptoms, regardless of breakthrough status, highlighting the need for reappraisal of mitigation strategies to prevent infection and potential development of autoimmune disease.

## Materials and Methods

### Study participants, National Institutes of Health toolbox, and PROMIS-57 data collection

**FIGURE 1.**
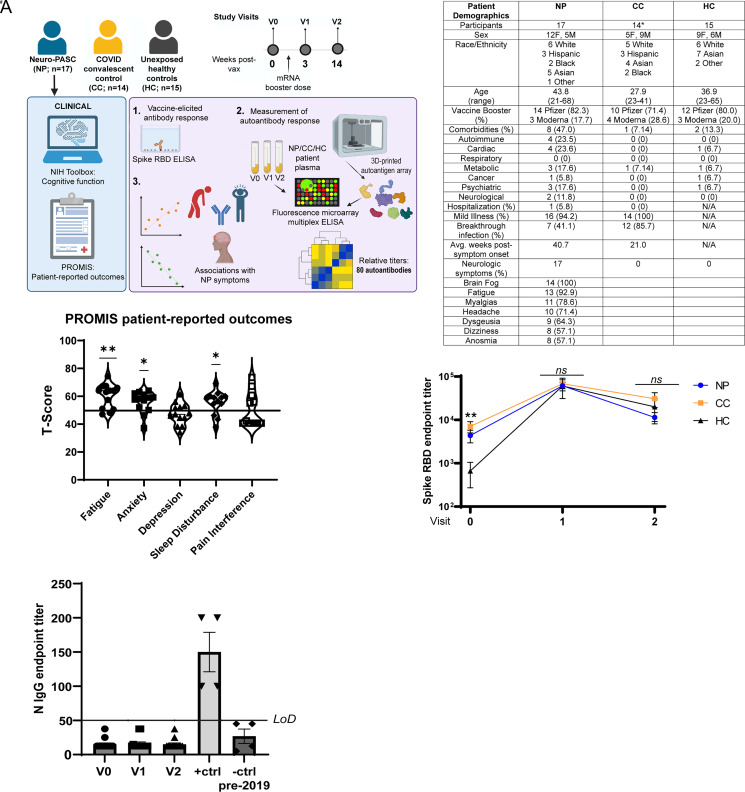
Study design, patient demographics, and vaccine-elicited antiviral humoral immunity. (**A**) Longitudinal study design. (**B**) Study subject demographics. (**C**) PROMIS-57 patient-reported outcome survey data for NP. Horizontal black line represents the U.S. national average T score of 50; *p* values are relative to demographic-matched U.S. national average by one sample *t* test. *p < 0.05; **p < 0.01. (**D**) mRNA vaccines elicit similar levels of anti-Spike RBD Abs in NP, CC, and HC. (**E**) HC remained unexposed to SARS-CoV-2 throughout the study as determined by anti-N IgG ELISA. +ctrl are NP with PCR-diagnosed SARS-CoV-2 infection; -ctrl refers to pre-2019 patient sera. ^#^Detailed neurologic symptom information was only collected for 14 of 17 patients with PASC; 3 of 17 reported generalized persistent neurologic symptoms. LoD, limit of detection.

Adult patients evaluated at our Neuro-COVID-19 clinic Northwestern Memorial Hospital were consented and enrolled from August 2021 through March 2022, including 17 NP with documented PCR^+^, rapid Ag^+^, or seropositive IgG results for SARS-CoV-2 prior to vaccination. All NP had lingering neurologic symptoms lasting >12 wk, and detailed symptom information was collected from 14 of 17 patients. In parallel, we recruited 12 CC from the surrounding community who tested either PCR^+^ or seropositive for SARS-CoV-2 before booster vaccination but had no lingering symptoms lasting >4 wk and 17 healthy control subjects (HC) who tested PCR^−^ for SARS-CoV-2 and were also seronegative for IgG against SARS-CoV-2 Spike receptor-binding domain (RBD) prior to vaccination. Two HC contracted SARS-CoV-2 breakthrough infections postvaccination but exhibited no lingering symptoms by visit 2 (3.5 mo postvaccination) and were included as HC at visit 0 but as CC at visit 2. All subjects had received either the Pfizer or Moderna mRNA vaccine prior to enrollment. All study subjects remained living throughout the period of observation. Heparinized blood samples were collected one time from each subject at an average of 21–40 wk post-symptom onset (as in Fig. 1B). NP (13 of 17) completed a cognitive function evaluation in the clinic coincident with or near the date of their blood sample acquisition with the National Institutes of Health (NIH) Toolbox version 2.1 instrument, including assessments of processing speed, attention, executive function, and working memory. PROMIS-57 (Patient-Reported Outcomes Measurement Information System) patient-reported quality of life assessments were administered to NP at the time of clinic visit. The results are expressed as T-scores, with a score of 50 representing the normative mean/median of the U.S. reference population and an SD of 10.

### Plasma collection

Venous blood (30 ml) from study volunteers was collected in blood collection tubes containing sodium heparin from BD Biosciences. Whole blood was layered on top of 15 ml of Histopaque 1077 (Sigma-Aldrich) in 50-ml Leucosep blood separation tubes (Greiner Bio-One) and spun at 1000 × *g* for 18 min at room temperature. Plasma was collected and stored at −80°C until further use.

### IgG Spike RBD and Nucleocapsid ELISA

Spike RBD and nucleocapsid-specific total Ab titers were measured by ELISA as described previously (8). In brief, 96-well flat-bottomed plates were coated with Spike RBD or Nucleocapsid protein for 48 h at 4°C. Blocking was performed for 4 h at room temperature. Serum (6 µl) was added to 144 µl of blocking solution in the first column of the plate, 1:3 serial dilutions were performed until row 12 for each sample, and plates were incubated for 60 min at room temperature. Plates were washed, followed by addition of secondary Ab conjugated to HRP, goat anti-human IgG (H + L) (Jackson ImmunoResearch). Plates were developed with SureBlue substrate (SeraCare), and the reaction was stopped after 1 min with KPL TMB Stop Solution (SeraCare). Absorbance was measured at 450 nm using a SpectraMax Plus 384 plate reader (Molecular Devices). SARS-CoV-2 RBD and N protein used for ELISA were produced at the Northwestern Recombinant Protein Production Core by Dr. Sergii Pshenychnyi with plasmids produced under HHSN272201400008C and obtained from BEI Resources, National Institute of Allergy and Infectious Diseases, NIH: Vector pCAGGS containing the NR-52394 Spike RBD protein region or NR-52309 from the Wuhan strain of SARS-CoV-2, nucleocapsid gene NR-53507.

### Autoantibody profiling

Plasma IgG reactivities against 79 discrete autoantigens were assessed using a microfluidic Ag array available through the Microarray and Immune Phenotyping Core at the University of Texas Southwestern Medical Center as described previously (9). In brief, plasma was spotted in triplicate on a 16-pad FAST slide. Autoantibodies binding to arrayed Ags generated via three-dimensional printing and validated by the University of Texas Southwestern Microarray Core Facility were detected with cyanine 3–labeled anti-human IgG. Two internal control proteins (one human IgG, one mouse IgG) were run in four different concentrations as positive and negative controls. Arrays were scanned with the GenePix 4400A Microarray Scanner (Molecular Devices), and images in the array were converted to GenePix Report files with GenePix Pro 7.0 software (Molecular Devices). The averaged fluorescent signal intensity of each Ag was subtracted by local background and the PBS control signal and normalized to internal controls to obtain the normalized fluorescence intensity value. Raw data (Z scores) are provided in Supplemental Dataset 1.

### Quantification and statistical analysis

Statistical tests to determine significance are described in figure legends and conducted in Prism (GraphPad Software) and in R. Z scores for autoantibody heatmaps were calculated in R on the basis of the mean and SD of the HC signal for each Ab. The matrix correlating long COVID symptoms and autoantibody titers was created in R using corrplot. Clinical data were collected and managed using REDCap electronic data capture tools hosted at Northwestern University Feinberg School of Medicine. All error bars on figures represent values ± SEM.

### Study approval

This study was approved by the Northwestern University Institutional Review Board (IRB STU00212583). Informed consent was obtained from all enrolled participants. Samples were deidentified before banking.

## Results

We enrolled a total of 46 study participants recruited from the Neuro-COVID-19 outpatient clinic at Northwestern Memorial Hospital or from the surrounding Chicago area. These included 17 NP (confirmed RT-PCR^+^ or rapid Ag^+^ before or after vaccination or seropositive prior to vaccination) recruited between August 2021 and March 2022 and who had neurologic symptoms lasting at least 12 wk but up to 88 wk postinfection. Among those, 16 (94.1%) had mild acute disease without pneumonia or hypoxia not requiring hospitalization. We additionally recruited 12 CC (RT-PCR^+^ or rapid Ag^+^ pre- or postvaccination) without symptoms persisting for more than 4 wk from onset, all of whom had mild acute disease. Two HC contracted breakthrough infections after vaccination and were included in the CC group at visit 2 because they had no lingering symptoms 3 mo postinfection. As older individuals tend to have elevated autoantibody levels even in the absence of autoimmune disease because of aging (10, 11), the CC group was intentionally selected to be younger than the NP group to limit the effects of older age on autoantibody titers preinfection. Finally, 15 HC with no prior history of SARS-CoV-2 exposure or autoimmune disease (RT-PCR^-^ and seronegative) were also included. Plasma samples were collected preboost and at 3 and 14 wk postboost to compare longitudinal vaccine-elicited and autoantibody responses (study design, Fig. 1A).

Neuro-PASC symptoms were consistent with those previously reported by our group, including brain fog, fatigue, headache, and myalgia. Plasma samples were collected at an average of 21–40 wk postinfection, with 41% of NP and 85.7% of CC having contracted their only SARS-CoV-2 infection as a breakthrough infection after receiving their primary COVID-19 vaccine series (Fig. 1B). NP reported quality of life impairment in multiple domains, including fatigue, anxiety, and sleep disturbance, which were significantly higher than the demographic-matched U.S. national average (Fig. 1C) and CC from previous studies (12).

Endpoint titer ELISA was used to measure vaccine-elicited Ab responses over time. IgG responses to the Spike receptor binding domain from the Wuhan strain of SARS-CoV-2 were significantly higher preboost in NP and CC with prior SARS-CoV-2 exposure, but all groups exhibited similar titers at 3 wk and 14 wk postboost (Fig. 1D). All HC were seronegative for anti-Nucleocapsid IgG, confirming that they were not exposed to SARS-CoV-2 prior to the study (Fig. 1E). These data demonstrate that mRNA vaccines elicit similar anti-Spike RBD IgG responses at 14 wk in most individuals, regardless of prior SARS-CoV-2 exposure.

**FIGURE 2.**
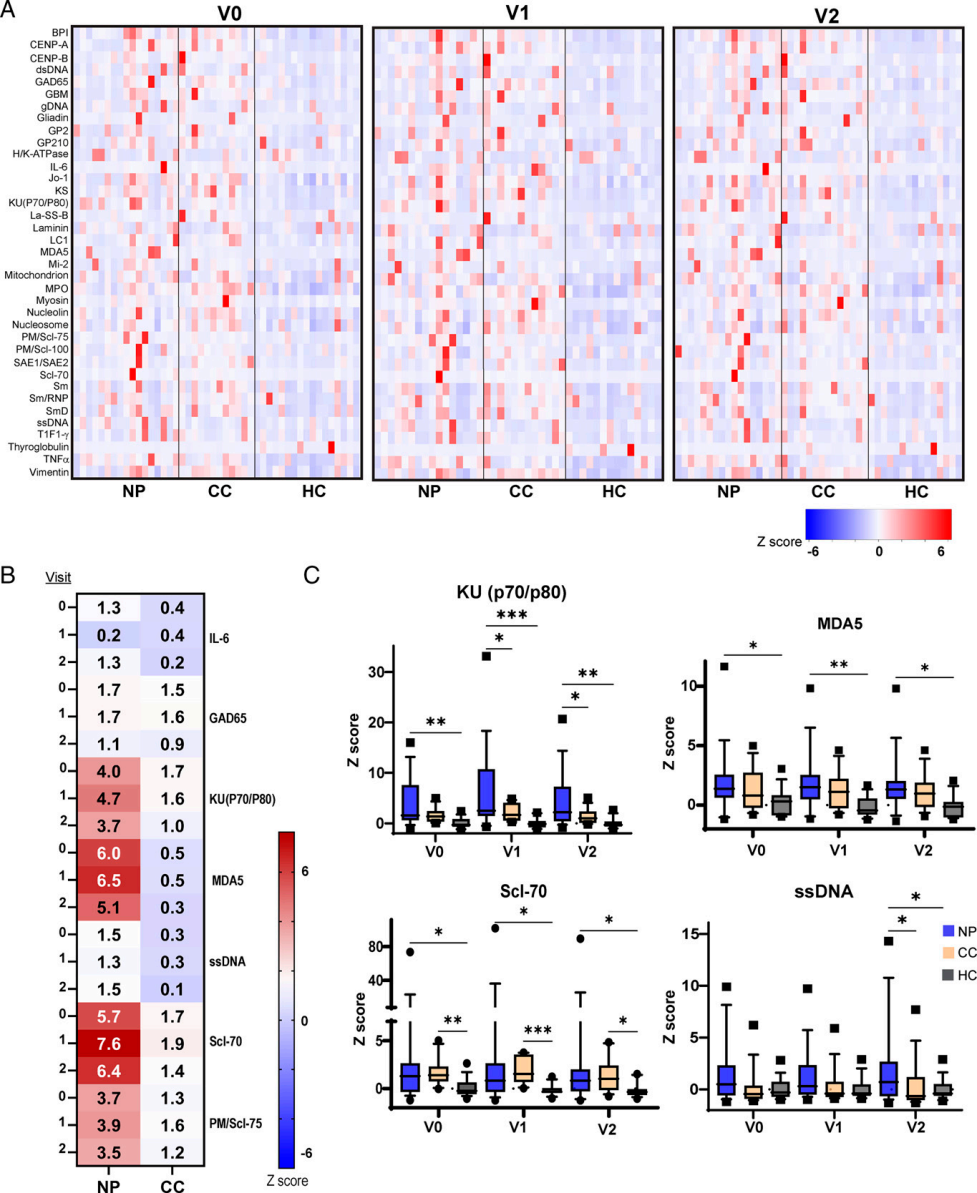
SARS-CoV-2 infection provokes sustained autoantibody elevation in both subjects with and without PASC. (**A**) Autoantibodies with diverse specificities are elevated in NP and CC compared with HC at all study visits. (**B** and **C**) Autoantibodies associated with SLE, systemic sclerosis, and inflammatory myopathy are particularly elevated in NP (*n* = 17) over CC (*n* = 14) and HC (*n* = 15). *p < 0.05; **p < 0.01; ***p < 0.005 by Kruskal-Wallis multiple comparisons test with uncorrected Dunn posttest. Multiple comparisons were corrected for by controlling the false discovery rate (0.05) in (C). Z scores were calculated in R relative to the mean and SD of the HC group for each autoantibody.

We next detected autoantibody responses in study subjects before and after their mRNA vaccine booster dose. Plasma samples were collected at the indicated time points and screened for the presence of 79 autoantibodies using the IgG Autoantigen Microarray Super Panel (Selected data in Fig. 2A, heatmap of all data in Supplemental Fig. 1, list of targets in Supplemental Table I). Data are shown as heatmaps of Z scores relative to the mean and SD for each autoantibody within the HC group, as shown in other studies investigating autoantibody levels in long COVID (5). Autoantibody levels for individual HC participants were also plotted relative to the groupwise HC Z scores because they represented baseline autoantibody levels in the absence of SARS-CoV-2 infection. Most NP had elevated autoantibody levels compared with HC at visits 0, 1, and 2 (Fig. 2A; raw data in Supplemental Table II). Autoantibodies implicated in inflammatory myopathy (MDA5), systemic sclerosis (Scl-70, PM/Scl-75), and systemic lupus erythematosus [SLE; KU(P70/P80), ssDNA] were particularly enriched in NP when compared with CC or HC and were most highly elevated at visit 2 postboost (Fig. 2B, 2C). Most autoantibody titers were not significantly correlated with age, sex, race, or the presence of autoimmune comorbidities, although elevated LPS, myelin basic protein, nucleolin, proliferating cell nuclear Ag, and Ro/SS-A autoantibody titers were correlated with increased age (Supplemental Fig. 2A, 2B). However, these autoantibody levels did not significantly differ between the NP and CC groups. Overall, these results suggest that even mild prior infection with SARS-CoV-2 can enhance humoral autoreactivity in people exposed to COVID-19, as seen with autoantigen profiling in other studies (13).

**FIGURE 3.**
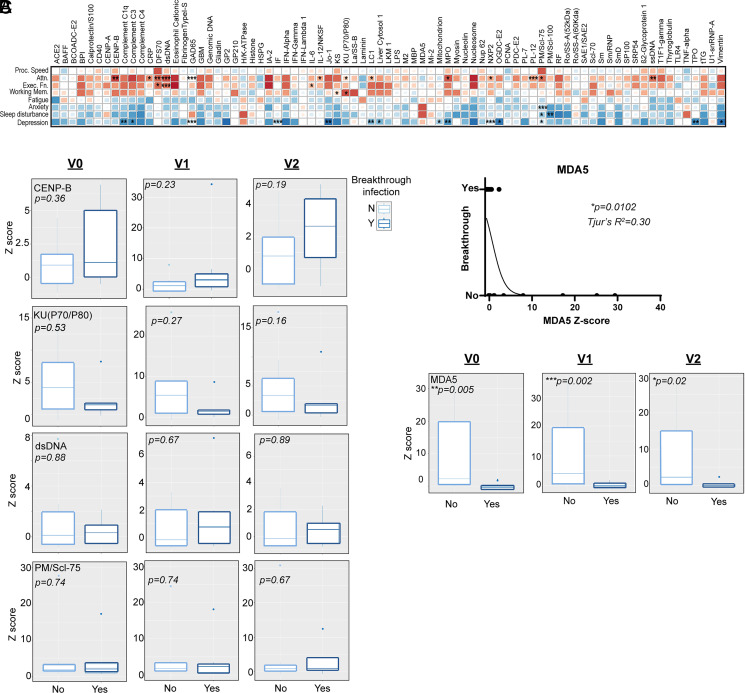
Elevations in SLE-associated autoantibodies significantly correlate with neuro-PASC cognitive dysfunction and symptom severity but not breakthrough infection status. (**A**) Correlations (dark red = negative or −1, dark blue = positive or +1) between cognitive performance and neurologic/psychiatric symptom severity and autoantibody responses in NP. Most significant associations were found between elevated SLE-associated autoantibodies and lower cognitive function/higher symptom severity. Attn., attention; Exec. Fn., executive function; Proc. Speed, processing speed; Working mem., working memory. (**B**) Breakthrough infections after vaccination did not result in lower levels of autoantibodies associated with worse cognitive and neurologic symptoms in NP. (**C**) Of the 79 measured analytes, MDA5 was the only autoantibody significantly enriched in NP who had primary and not breakthrough infections (logistic regression, top panel). *p < 0.05, **p < 0.01, ***p < 0.005 by Spearman correlation (A), Wilcoxon rank-sum test (B), two-way ANOVA with correction for multiple comparisons by false discovery rate (0.05; C, bottom panels), or simple logistic regression (C, top panel).

Higher autoantibody levels in NP were correlated with poor cognitive performance as determined by the NIH Toolbox cognition battery, which measures cognitive domains known to be important for intellectual function, independence, and scholastic/workplace success. High titers of the GAD65 autoantibody linked to cerebellar ataxia, stiff-person syndrome, and chronic epilepsy, as well as type 1 diabetes, were strongly associated with poor results in the attention module and the presence of depression, whereas higher titers of the myositis Ab PM/Scl-75 correlated with low attention scores and elevated psychiatric and sleep disturbances, among other significant findings (Fig. 3A). NP with preexisting autoimmune diseases were more likely to have elevated anti–C-reactive protein, fibrinogen I, IFN, and Sm/RNP Abs (Supplemental Fig. 2A), but these were not associated with symptom severity (Fig. 3A). No significant differences in symptom-associated autoantibodies were found between patients with and without preexisting autoimmune disease (Supplemental Fig. 2B). These results demonstrate that autoantibody elevations linked to neuronal dysfunction, SLE, and systemic sclerosis in NP correlate with cognitive dysfunction and symptom severity.

Patients who contract breakthrough infections (i.e., those who become infected with SARS-CoV-2 after vaccination) have a 15% lower risk of developing postacute cardiac, metabolic, neurologic, and musculoskeletal symptoms than those infected prior to vaccination (7). On this basis, we theorized that autoantibody responses may be lower in NP with breakthrough rather than primary infections. In contrast, we found that autoantibody responses were similar in NP, regardless of breakthrough status, particularly with respect to autoantibodies associated with cognitive symptom severity (Fig. 3B), although caution should be taken when interpreting these data because of small sample sizes. One exception was MDA5, which was enriched in nonbreakthrough NP throughout the course of the study and is associated with inflammatory myopathy and suppression of antiviral immunity (14) (Fig. 3C). No other significant correlations between autoantibody titers and breakthrough status were found using logistic regression analyses.

Our study demonstrates that mild acute SARS-CoV-2 infection can lead to elevated humoral immunity, particularly with respect to rheumatic disease–associated autoantibodies, with worse outcomes for patients with persistent neurologic symptoms of long COVID. We additionally show that vaccination does not decrease autoimmunity elicited by subsequent breakthrough infections. Altogether, these data demonstrate that autoimmunity can be a consequence of mild COVID-19 that cannot be mitigated by vaccination alone.

## Discussion

Up to 30% of people infected by SARS-CoV-2 experience persistent symptoms, and it is estimated that over 45 million adults in the United States are affected by long COVID/PASC. Although the precise mechanisms remain unclear, there is increasing evidence implicating humoral autoimmunity in the pathogenesis of neuro-PASC after severe SARS-CoV-2 infection (15). However, whether mild acute COVID-19 impacts autoimmunity and how this differs upon breakthrough infection after vaccination have not been examined. We aimed to fill this knowledge gap by longitudinally examining how infection and vaccination affect autoantibody responses to 79 Ags in NP, CC, and HC.

Over 94% of NP and 100% of CC in our study presented with mild acute disease. Despite this, 88% of NP and 85% of CC had significant elevations of multiple autoantibodies postinfection, despite the fact that CC were young and had few preexisting comorbidities. Studies to date have focused on the links between autoimmunity and acute disease severity because higher inflammation during severe disease can lead to bystander activation of autoreactive B and T cells or epitope spreading caused by excessive tissue damage (16). Indeed, the inability to resolve autoantibody elevation up to 1 y after severe acute infection has been linked to the persistence of fatigue, dyspnea, and other symptoms (17). In contrast, our data show that mild infection can lead to similar autoimmune consequences.

There is evidence that mild coronavirus disease led to persistent symptoms, including cognitive impairment, muscle weakness, and dysautonomia, during the Russian pandemic of 1889–1891 (18), and 83% of COVID-19 convalescents with mild or severe acute disease were found to have latent autoimmunity in the current pandemic (19), demonstrating that elevated autoantibody titers may not always result in immediate symptoms. It is possible that mild SARS-CoV-2 infection constitutes an environmental trigger that can induce latent autoimmunity in some people because of epitope spreading predicted to occur after COVID-19 in bioinformatic studies (20), which eventually manifests as symptomatic autoimmunity in NP (21). Indeed, large retrospective cohort studies have shown that SARS-CoV-2 infection of any severity significantly increases the risk of developing rheumatoid arthritis, SLE, systemic sclerosis, and other rheumatic autoimmune diseases (22).

Our data also suggest that the development of latent autoimmunity in COVID-19 convalescents may put them at risk for PASC with recurrent SARS-CoV-2 infections. It has previously been shown that individuals were at a much higher risk for postacute sequelae, including neurologic and musculoskeletal manifestations, after multiple infections than those infected only once (3). It is possible that we identified such pronounced elevations of autoantibodies after mild acute disease in contrast to other reports because our samples were collected at least 5 mo and up to 1.5 y after acute infection as opposed to within 3 mo of infection. This highlights the need for longitudinal studies to define autoimmune correlates and development of symptoms in patients with PASC and without PASC.

Autoantibodies associated with SLE and other rheumatic autoimmune diseases were more elevated in NP than CC and significantly correlated with lower cognitive performance and higher symptom severity, particularly with respect to depression. Interestingly, high levels of the dermatomyositis Ab MDA5 were found in patients with COVID-19 with severe acute disease who eventually died of infection (23). In our study, we detected significant elevations in MDA5 autoantibodies solely in NP and not CC. MDA5 is a cytosolic RIG-I-like receptor that senses viral RNA and leads to the production of type I IFNs (14). It is well established that patients with moderate to severe acute COVID-19 have elevated autoantibodies against type I IFNs (24), but our data suggest that autoantibodies against MDA5 upstream of type I IFN production may be involved in the pathogenesis of PASC. MDA5 levels were highly correlated with having a primary and not breakthrough SARS-CoV-2 infection, suggesting that vaccination may help prevent MDA5 autoimmune responses or that specific SARS-CoV-2 strains may induce different varieties of autoantibody responses. NP with primary infections were mostly infected during the Delta wave, and those with breakthrough infections were infected in the Omicron wave. Further studies are needed to determine whether SARS-CoV-2 can induce anti-MDA5 Abs as an immune evasion strategy during persistent infection, which has also been linked to PASC (25, 26).

Previous studies have convincingly shown that SARS-CoV-2 mRNA vaccines do not induce autoantibody responses (27), but they did not assess whether vaccination prior to breakthrough infections can limit autoimmunity in COVID-19 convalescents. We found that booster vaccine doses did not decrease autoantibody responses in patients with breakthrough infections, suggesting that vaccination cannot be used to combat autoimmunity resulting from exposure to SARS-CoV-2.

These data highlight the need for additional strategies to decrease the spread of SARS-CoV-2 such as the development of vaccines that can prevent infection rather than decrease severity of acute disease alone. Longitudinal studies are also needed to determine whether autoimmune responses in COVID-19 convalescents may lead to neuro-PASC symptoms after recurrent SARS-CoV-2 infections. In conclusion, our study emphasizes the need for continuous development and implementation of public health strategies to decrease the spread of SARS-CoV-2 in the community to limit the burden of autoimmune disease.

## Supplementary Material

Supplemental Material (PDF)

Supplemental Material (CSV)

Supplemental Material (CSV)
