# Supplementary material for: Mild Primary or Breakthrough SARS-CoV-2 Infection Promotes Autoantibody Production in Individuals with and without Neuro-PASC
Source: Immunohorizons. 2024 Aug 26;8(8):577–85. doi: 10.4049/immunohorizons.2400033 (PMC11374748; doi:10.4049/immunohorizons.2400033)
Supplement: Supplemental Material (PDF) [file IH_2400033_Supplemental_1.pdf]

Supplemental Figures

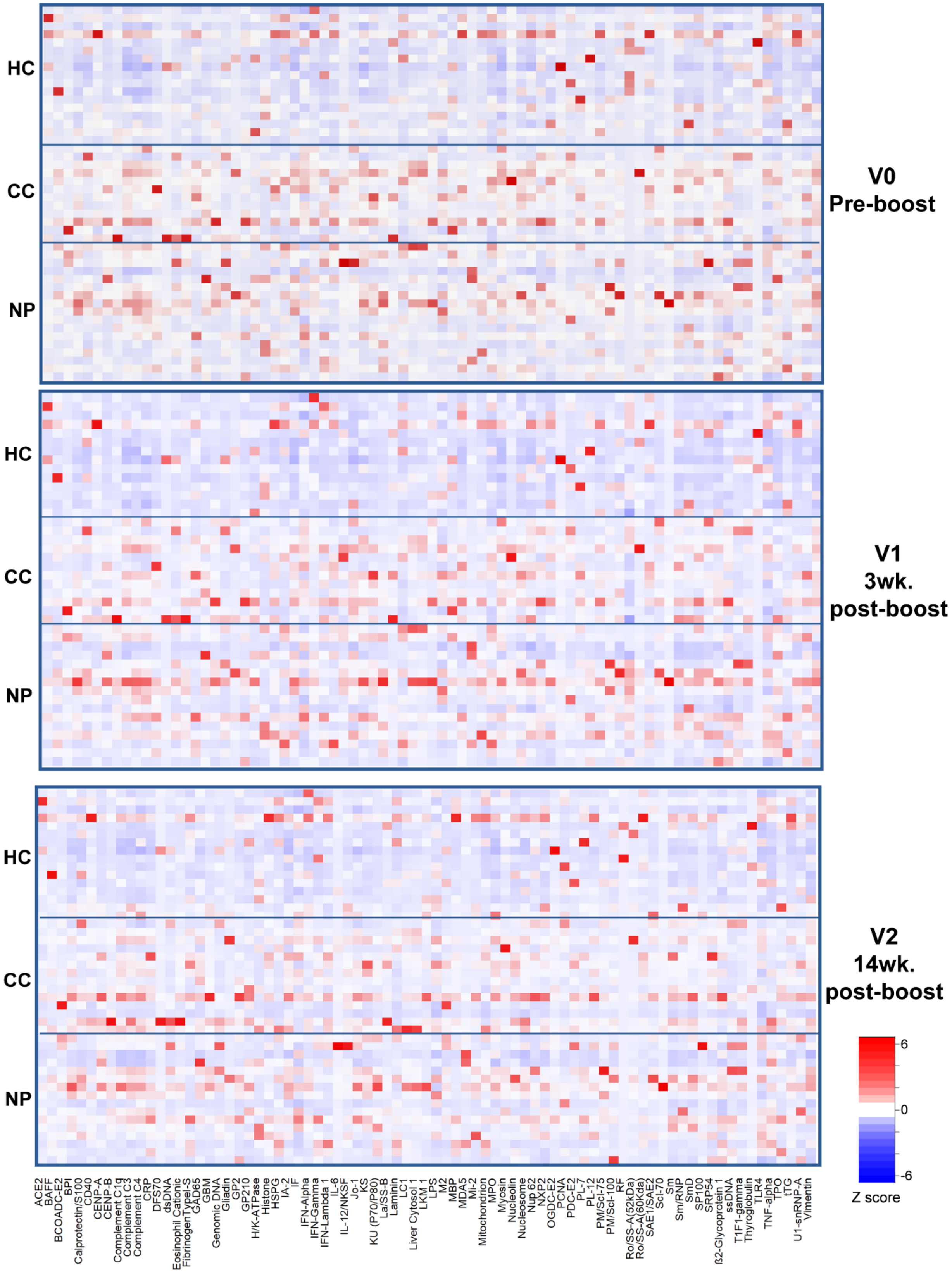

Figure S1: Heatmap of 79 autoantibodies measured in microarray.

Z scores were calculated in R based on the mean and standard deviation of the healthy control (HC) group at V0 for each autoantibody.

A.

	Autoantibody	p value	Z score	Spearman r
Sex: M v. F	None significant	NA	NA	NA
Age	LPS	0.03	NA	-0.39
	MBP	0.03		-0.39
	Nucleolin	0.01		-0.45
	PCNA	0.002		-0.54
	Ro/SS-A	0.03		-0.4
Race:				NA
White	tTg	0.01	2.5	
	Vimentin	0.02	2.3	
Black	NA			
Hispanic	NA			
Asian	tTg	0.04	2.1	
Autoimmune Comorbidities: 4Y/13N (RA, Hashimoto's, autoimmune GERD)	CRP	0.008	1.7	NA
	Fibrinogen Type 1	0.04	1.4	
	Interferon	0.01	1.9	
	Sm/RNP	0.02	1.5	

B.

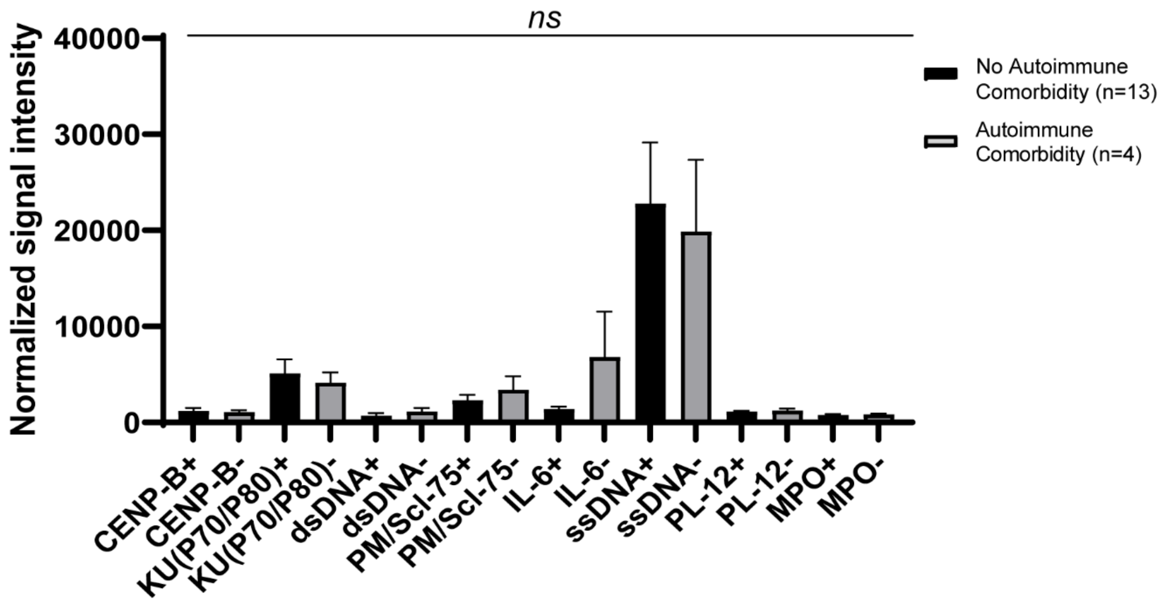

Figure S2: Correlations of demographics with autoantibody responses.

A). Logistic or linear regression of sex, age, and race (for all NP and CC subjects), and presence of autoimmune comorbidities (NP subjects only) with autoantibody Z scores. There were no significant correlations between demographics and autoantibodies linked to cognitive performance or neurologic symptom severity shown in Fig. 3A. B). Levels of autoantibodies linked with cognitive performance and symptom severity did not differ in Neuro-PASC patients with and without autoimmune comorbidities. *$p<0.05$, **$p<0.01$, ***$p<0.005$ by multiple linear regression (Age) or multiple logistic regression (Sex, Race, Autoimmune comorbidity; S2A).

Table S1: Autoantibodies screened in microarray

Protein	Autoantibody symbol
Aggrecan	Aggrecan
Alanyl-tRNA Synthetase (PL-12)	PL-12
Asparaginyl-tRNA Synthetase (KS)	KS
Bactericidal/permeability-increasing protein (BPI)	BPI
ß2-Glycoprotein 1	ß2-Glycoprotein 1
BCOADC-E2	BCOADC-E2
Cardiolipin	Cardiolipin
Centromere Protein A (CENP-A)	CENP-A
Centromere Protein B (CENP-B)	CENP-B
Chondroitin sulfate	Chondroitin sulfate
Collagen III	Collagen III
Collagen V	Collagen V
Complement C3	Complement C3
Complement C4	Complement C4
Complement component C1q receptor (C1q)	Complement C1q
Cytochrome c	Cytochrome c
Cytochrome P450 2D6 (LKM 1; ng)	LKM 1
DFS70	DFS70
DNA Topoisomerase I (Scl-70)	Scl-70
Double-stranded DNA (dsDNA)	dsDNA
Elastin	Elastin
Fibrinogen Type I-S	Fibrinogen Type I-S
Formiminotransferase Cyclodeaminase (LC1)	LC1
Genomic DNA	Genomic DNA
Gliadin	Gliadin
Glomerular Basement Membrane (GBM)	GBM
Glutamate Decarboxylase 65 kDa (GAD65; ng)	GAD65
GP2	GP2
GP210	GP210
Heparan sulfate proteoglycan(HSPG)	HSPG
Histidyl-tRNA Synthetase (Jo-1)	Jo-1
Histone	Histone
Human CD 40	Human CD 40
IA-2 (ICA 512)Insulinoma-associated protein (IA-2)	IA-2
Insulin	Insulin

Intrinsic Factor (IF)	IF
KU (P70/P80)	KU (P70/P80)
La/SS-B	La/SS-B
Laminin	Laminin
M2	M2
MDA5	MDA5
Mi-2	Mi-2
Myelin basic protein (MBP)	MBP
Myeloperoxidase (MPO)	MPO
Myosin	Myosin
Nucleolin	Nucleolin
Nucleosome	Nucleosome
Nup 62	Nup 62
NXP2 also known as MORC3	NXP2
OGDC-E2	OGDC-E2
PDC-E2	PDC-E2
PM/Scl 100	PM/Scl 100
PM/Scl-75	PM/Scl-75
Proliferating Cell Nuclear Antigen (PCNA)	PCNA
Proteinase 3 (PR3)	PR3
Proteoglycan	Proteoglycan
Ribosomal Phosphoprotein P0	P0
Ribosomal Phosphoprotein P2	P2
Ro/SS-A (52 kDa)	Ro/SS-A (52 kDa)
Ro/SS-A (60 Kda)	Ro/SS-A (60 Kda)
SAE1/SAE2	SAE1/SAE2
Sm	Sm
Sm/RNP	Sm/RNP
SmD	SmD
SmD1	SmD1
SP100	SP100
Fibrinogen IV	Fibrinogen IV
SRP54	SRP54
ssDNA	ssDNA
T1F1 gamma	T1F1 gamma
Threonyl-tRNA Synthetase (PL-7)	PL-7
Thyroglobulin	Thyroglobulin
Thyroid Peroxidase (TPO)	TPO
Tissue Transglutaminase (tTG)	tTG
TNF-alpha	TNF-alpha

U1-snRNP 68/70 kDa	U1-snRNP 68/70 kDa
U1-snRNP A	U1-snRNP A
U-snRNP B/B'	U-snRNP B/B'
Vimentin	Vimentin
Vitronectin	Vitronectin
